# Genetic Diversity of the Hemagglutinin Genes of Influenza a Virus in Asian Swine Populations

**DOI:** 10.3390/v14040747

**Published:** 2022-04-01

**Authors:** Takehiko Saito, Saki Sakuma, Junki Mine, Yuko Uchida, Basav N. Hangalapura

**Affiliations:** 1 National Institute of Animal Health, National Agriculture and Food Research Organization (NARO), Tsukuba 305-0856, Japan; sakumas438@affrc.go.jp (S.S.); minejun84032@affrc.go.jp (J.M.); uchiyu@affrc.go.jp (Y.U.); 2 MSD-Animal Health, 5831 AN Boxmeer, The Netherlands; basav.nagaraj@merck.com

**Keywords:** pig, respiratory disease, phylogeny, vaccine

## Abstract

Swine influenza (SI) is a major respiratory disease of swine; SI is due to the influenza A virus of swine (IAV-S), a highly contagious virus with zoonotic potential. The intensity of IAV-S surveillance varies among countries because it is not a reportable disease and causes limited mortality in swine. Although Asia accounts for half of all pig production worldwide, SI is not well managed in those countries. Rigorously managing SI on pig farms could markedly reduce the economic losses, the likelihood of novel reassortants among IAV-S, and the zoonotic IAV-S infections in humans. Vaccination of pigs is a key control measure for SI, but its efficacy relies on the optimal antigenic matching of vaccine strains with the viral strains circulating in the field. Here, we phylogenetically reviewed the genetic diversity of the hemagglutinin gene among IAVs-S that have circulated in Asia during the last decade. This analysis revealed the existence of country-specific clades in both the H1 and H3 subtypes and cross-border transmission of IAVs-S. Our findings underscore the importance of choosing vaccine antigens for each geographic region according to both genetic and antigenic analyses of the circulating IAV-S to effectively manage SI in Asia.

## 1. Introduction

Swine influenza (SI) is a respiratory disease caused by the influenza A virus of swine (IAV-S), which belongs to the genus *Alphainfluenza* virus in the family *Orthomyxoviridae*; IAV-S carries eight negative-strand segments of RNAs as its genome [1]. IAV-S is highly contagious and a major pathogen of the porcine respiratory disease complex (PRDC): morbidity can reach as high as 100% within a herd. SI causes flu-like symptoms in pigs similar to those of human influenza, including high fever, coughing, and depression; in addition, SI results in stunting and delayed weight-gain in fattening and finishing pigs [2,3,4,5]. Coinfection with *Mycoplasma* spp., porcine reproductive and respiratory syndrome virus (PRRS virus), porcine circovirus 2 (PCV-2), or other respiratory pathogens can exacerbate the symptoms of SI [2,6]. The pig industry in the United Kingdom loses approximately GBP 65 million per year due to SI [7]. In addition, asymptomatic infection with IAV-S has been widely recognized recently [8,9,10]. Overall economic losses due to IAV-S in the pig industry are very substantial [4,11,12].

**Figure 1:**
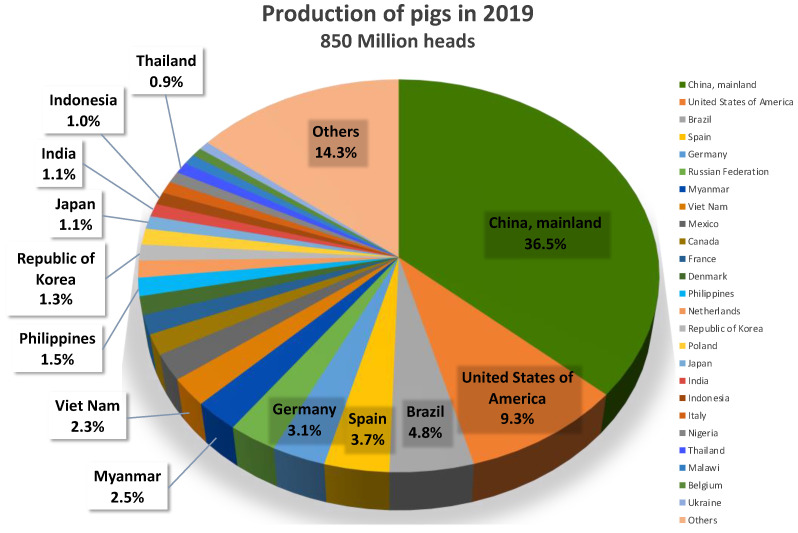
The top five pig-producing countries worldwide and the top nine pig-producing Asian countries. Data was obtained from FAOSTAT (http://www.fao.org/faostat/en/#data/QCL, accessed on 9 July 2021).

**Figure 2:**
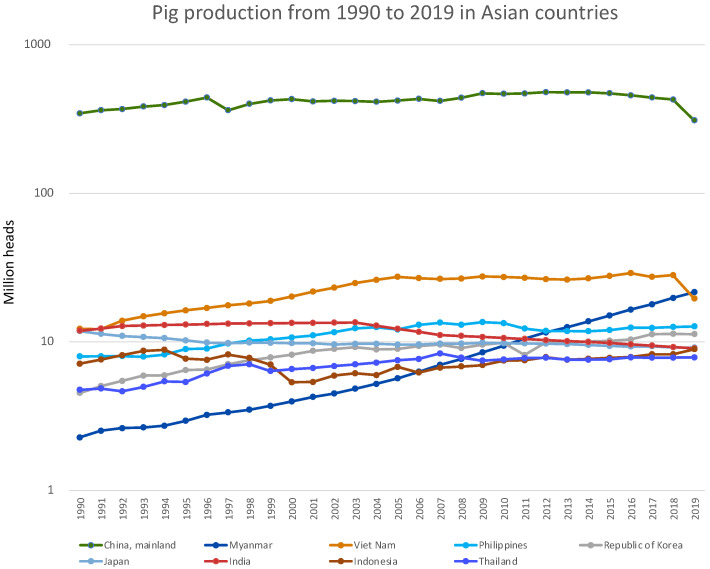
Trends in pig production from 1990 through 2019 for the nine Asian countries in Figure 1.

Half of all pigs worldwide are produced in Asian countries, with China as the top pig-producing country, accounting for 36.5% of worldwide pig production in 2019 [13]. In addition, Myanmar and Vietnam each accounted for more than 2% of overall pig production, followed by the Philippines, Korea, Japan, India, Indonesia, and Thailand among the Asian countries (Figure 1). In particular, the pig production industry in Myanmar has increased steadily over the last three decades, with a current market share that is 10 times what it was initially (Figure 2). However, the intensity of IAV-S surveillance varies among Asian countries [14,15,16], because SI is not a reportable disease and because SI has far less of an effect on the pig industry than other enzootic diseases in Asia, such as classical swine fever, foot-and-mouth disease, PRRS, and the recently emerged African swine fever.

Combined with robust hygiene practices, vaccination is one of the most effective control measures to reduce SI-associated economic loss at the farm level [4]. However, due to the error-prone nature of the viral RNA polymerase [17], non-synonymous substitutions in the hemagglutinin (HA) genes accumulate over time [14,18,19,20,21]. In particular, the accumulation of substitutions in the antigenic sites of the HA protein, a primary target of host neutralizing antibodies, can lead to antigenic drift from the ancestral strain [22,23,24,25]. The effectiveness of SI vaccines rests heavily on correct antigenic matching between viral strains circulating in the field and the vaccine strain. Although other factors including antigen content and type of adjuvant may influence vaccine efficiency [26,27,28], antigenic match is the leading determinant in vaccine efficacy [29,30,31]. Therefore, maintaining the efficacy of commercially available SI vaccines requires continued monitoring of the antigenicity of circulating strains through hemagglutination-inhibition or neutralizing tests—followed by challenge studies, if necessary—and subsequent updating of vaccine composition [15,32,33]. However, in many countries, updating the composition of a vaccine involves a cumbersome registration process that is almost equal in effort to registering a new vaccine. Unlike that for human seasonal influenza vaccines, the process for updating SI vaccine composition hinders a timely update [33]. An exception to this drawback is the autogenous vaccines available in the United States; these herd-specific vaccines are designed specifically against an IAV-S isolate from a herd within the pig production system where the vaccine is used [3,34,35].

IAVs-S of the H1N1, H1N2, and H3N2 subtypes are currently circulating worldwide. The established clade classification system for the HA gene of H1 subtype IAVs-S recognizes three major lineages—1A, 1B, and 1C—and their sub-lineages [14]. Clade 1A used to be called ‘classical IAVs-S’, which share a common ancestor with Spanish Flu [36]; genes derived from human seasonal IAVs since 2009 (i.e., H1N1pdm) are recognized as sub-clade 1A.3.3.2. Clade 1B consists of genes that originated from human seasonal H1 IAVs before H1N1pdm, including the so-called Russian Flu circulating from 1977 to 2009 in humans. Viruses in clade 1C originated from the Eurasian avian (EA) influenza virus lineage, which has been endemic in the European pig population since the late 1970s [37]. In contrast, although reverse-zoonotic (i.e., human to pig) transmission of human seasonal H3 IAVs-S occasionally has occurred in different parts of world [18,21,24,38,39,40,41,42], no definitive H3 HA clade classification system has yet been established.

Neither the nucleotide nor amino acid sequence of the HA gene of IAVs-S can be used alone to infer the antigenic similarity [32] or efficacy of vaccines against the IAVs-S circulating in a country [27]. However, understanding the genetic diversity and evolution of IAVs-S provides pivotal information for designing effective SI vaccines for Asian countries, where SI vaccines are less recognized as a tool for controlling SI. For example, IAV-S vaccine coverage in Japan is estimated at <10% [18]. Despite the many studies regarding the genetic diversity of the HA genes of IAVs-S in individual countries, including China [20,43,44,45,46,47,48], Japan [18,23,25,49,50,51,52,53], Korea [5,54,55,56,57], Thailand [19,21,22,58,59], and Vietnam [42,60,61,62], the evolutionary relationship among Asian IAV-S isolates has not been examined in detail. Therefore, we phylogenetically reviewed HA sequence data obtained from the GISAID EpiFlu Database to clarify the cross-regional and national characteristics of the genetic diversity of the HA genes of IAVs-S in Asian countries, with particular focus on the IAV-S sequences generated during the last decade (i.e., 2010 through 2020).

## 2. H1 Subtype IAVs-S

**Figure 3:**
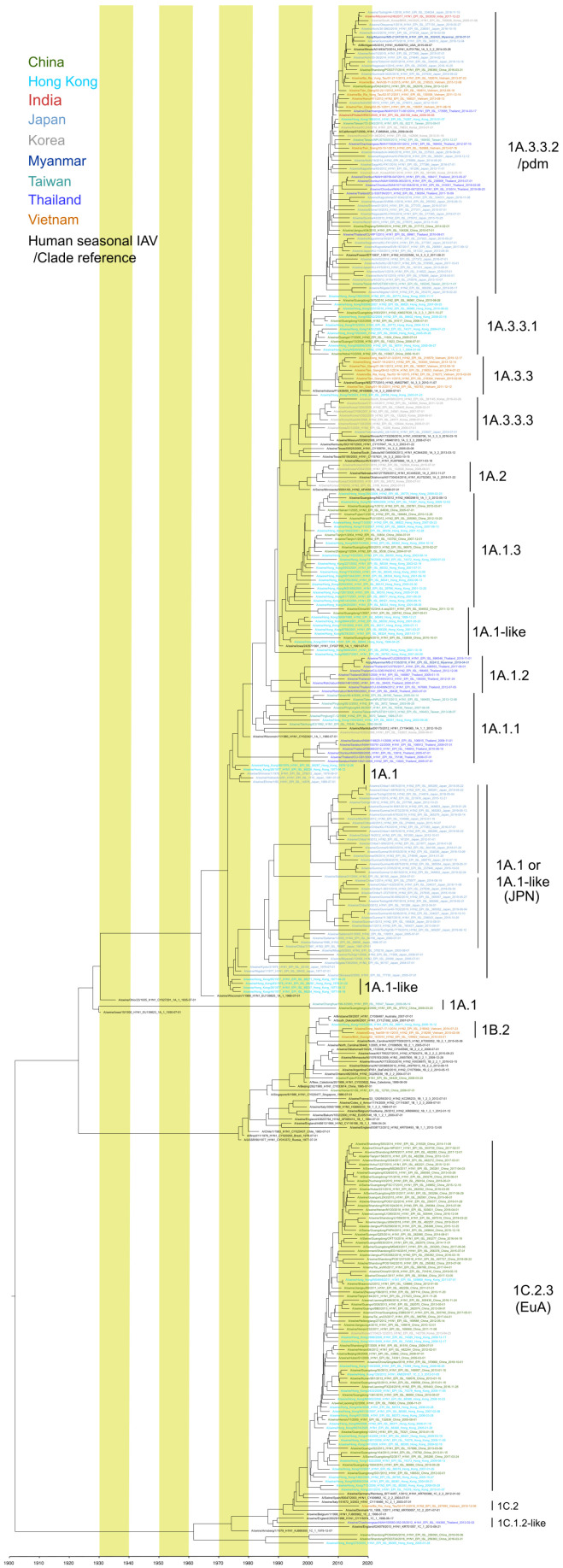
Maximum clade credibility (MCC) phylogenetic tree of the HA genes of Asian H1 IAVs-S. All available H1 HA gene sequences with complete HA coding regions from IAVs-S isolated in the nine Asian countries in Figure 2 were downloaded from GISAID on 8 July 2021. Sequence data from the Philippines were unavailable at the time of the download. Sequence data for each country or region underwent cd-hit clustering [69] (threshold, 98.5% identity) to reduce the number of sequences and increase visualization. ‘China’ refers to mainland China, and Hong Kong and Taiwan were considered as separate regions because they differ in pig trade and other livestock-related practices that might affect the prevalence of IAV-S. For the calculation, when only the year was available as the isolation date from GISAID, 1 July was used as the tentative isolation date; when only the year and month were available, the 15th was used as the day. This tree was constructed to illustrate the overall evolutionary pathway of Asian IAVs-S, not for precise calculation of the divergence time. Various the human seasonal IAVs and IAVs-S from other countries have been included as clade indicators.

Several subclades of H1 IAVs-S have cocirculated in Asian countries since 2010 (Table 1). Similar to the situation in other parts of the world [63,64,65,66,67], reverse-zoonotic transmission of H1N1pdm clade 1A.3.3.2, has occurred in China, Hong Kong, India, Japan, Korea, Myanmar, Taiwan, Thailand, and Vietnam; some of these 1A.3.3.2 viruses reassorted with the IAVs-S strains previously circulating in those countries and regions [18,19,20,22,43,44,49,52,58,60,68]. The precise geographic location where H1N1pdm (clade 1A.3.3.2) originated in swine remains unknown. However, the phylogenetic analysis of H1N1pdm strains isolated from Asia suggests that several incidents of the reverse-zoonotic transmission of H1N1pdm might have occurred in various countries (Figure 3). However, a lineage originating from any of these transmissions has not become the predominant IAV-S in any of these countries or regions. Although, clade 1A.3.3.2 is common throughout Asia, the prevalence of the other clades differs substantially from one country or region to another.

In China and Hong Kong, viruses belonging to clades 1A.3.3.1 and 1A.1.3 were isolated in the early 2000s [43,70]. Recent intensive surveillance in mainland China and Hong Kong has revealed the predominance of the 1C.2.3 lineage, which derived from EA-like IAVs-S, during 2011 to 2018 [43,45,46] followed by 1A.3.3.2. Within the 1C.2.3, antigenic drift was apparent between isolates in the early 2010s and the late 2010s in mainland China. They showed low cross reactivity in the hemagglutination-inhibition test to the antiserum raised against the human H1N1pdm strain isolated in 2015 [46]. Clearly, antigenicity differs between the 1C.2.3 isolates from 2011 to 2013 and those from 2016 to 2018.

In Vietnam, clades 1A.3.2.2, 1A.3.3 and 1B.2 appeared to be codominant [61]. During 2010 through 2015, 1A.3.3 strains circulated in southern Vietnam, whereas 1B.2 viruses were isolated in both the north and south. Although the 1C.2 clade was not well established throughout Vietnam, 1C.2 viruses—represented by A/swine/Ba Ria Vung Tau/02-07-2/2016—were isolated in southern Vietnam [62]. Transborder introduction of 1C.2 viruses into Vietnam from Europe was suggested, given that the farm where the 1C.2 viruses were isolated had purchased the pigs from another farm that imported pigs from Europe [62].

In Korea, viruses belonging to clade 1A.3.3.3 appeared as early as in 2004, were considered to originate from gamma-lineage strain(s) that circulated in the United States [5], and circulated throughout Korea until 2016. Furthermore, the 1A.2 viruses that circulated in the United States and Mexico were reported in Korea, however, the most recent strain of this clade was reported in 2010. IAVs-S of clade 1C.2.3 closely related to those circulating in Hong Kong were reported in Korea in 2013 but no later [57]. Pig importation has been suggested as the mechanism through which multiple IAVs-S from abroad have been introduced into Korea.

In Thailand, viruses belonging to 1A.1.2 and 1A.1.1 cocirculated in the 2000s [21,71,72], but only members of 1A.1.2 were reported during the 2010s [19,22]. Because sequence data regarding the 1A.1.2. viruses in the 2010s were obtained mainly through the longitudinal surveillance and the studies that focused on only a few farms, whether this clade became dominant throughout the Thai pig population is unknown. It is noteworthy that a 1A.1.2 isolate from Myanmar—A/pig/Myanmar/MS-21135-HA/2018 [68]—is closely related to A/swine/Thailand/CU22630/2018, indicating the transborder movement of IAVs-S between the neighboring countries. EA-like IAV-S belonging to 1C.1.2 was isolated from a farm in Thailand in 2012 [73], but no further reports of this clade have been made.

In Japan, the 1A.1-like clade has become established as a unique clade consisting exclusively of Japanese isolates since the 1980s [18,49,74]. Except for 1A.3.3.2 viruses originating from H1N1pdm strains, the IAVs-S in Japan has evolved as a single major clade since its first recognition in 1977 [50]. This single clade diverged into two sub-clades in the mid-1980s, both of which are circulating in Japan currently [18]. Whether this genetic divergence is indicative of the antigenic divergence has not yet been studied. In addition, although a virus belonging to 1A.3.3.3—A/swine/YokohamaAQ(US)/1/2014—was isolated from an imported pig under quarantine at a national Animal Quarantine Station in Japan, further dissemination of this clade in Japan has not been recognized.

## 3. H3 Subtype IAVs-S

Several clades of IAVs-S harboring the HA gene of human seasonal H3 HA lineages have been observed in Asian countries during the last decade. A/Bin Duong/03_06/2010 (BD)-like strains have been isolated in Vietnam [42,61], China, and Hong Kong [43]. The BD-like strains obtained in Vietnam originated from a human seasonal H3N2 strain that circulated between 2004 and 2005 [42]. An antigenic comparison of A/Bin Duong/03_06/2010 with a human H3N2 strain isolated in 2004 (a possible ancestor of A/Bin Duong/03_06/2010) and a human strain isolated in 2009 revealed considerable antigenic differences among these viruses [23], thus demonstrating independent antigenic drift in the pig population among viruses with a common origin. Although not directly related to BD-like strains, Japanese IAVs-S—represented by A/swine/Osaka-C/12-20/2008—similarly were shown to originate from a human seasonal H3N2 strains circulating around 2004 [23]. However, a descendant of these strains has not become the predominant strain in Japan.

In Japan, viruses carrying the HA gene of human seasonal H3N2 strains circulating during the 1999–2000 season—represented by A/swine/Kagoshima/37-7201/2019—have become established during the last decade [18,23,53,74]. These H3 subtype IAVs-S have been isolated in various regions of the country since 2000, suggesting dissemination of this clade of IAVs-S throughout Japan. Although IAVs-S with an HA of similar origin—represented by A/swine/Korea/CAS05/2004—have been isolated in Korea [5], they have not become established there.

**Figure 4:**
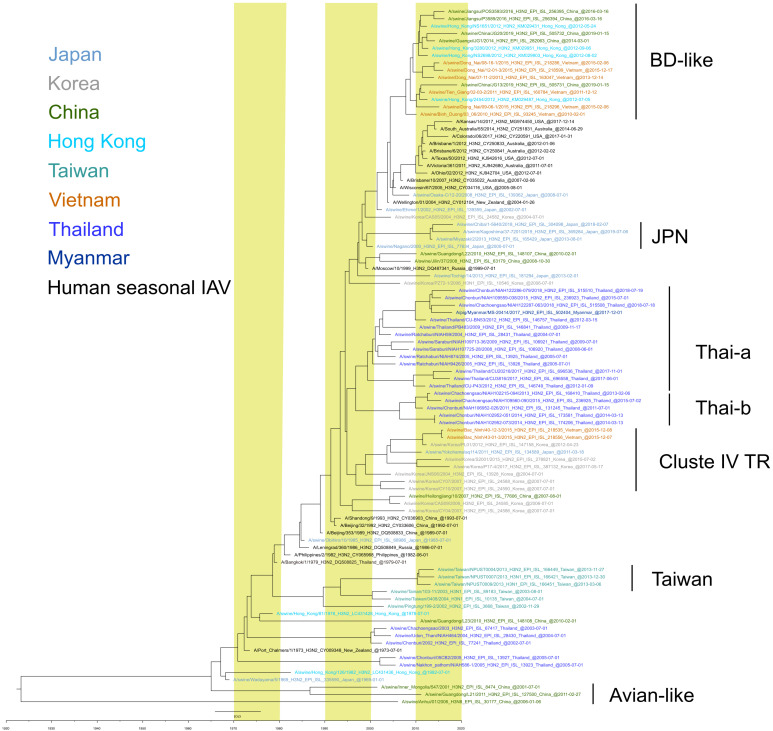
Maximum clade credibility (MCC) phylogenetic tree of the HA genes of H3 subtype Asian IAVs-S. All available H3 HA gene sequences with complete HA coding regions from IAVs-S isolated in the nine Asian countries in Figure 2 were downloaded from GISAID on 8 July 2021. Sequence data from the Philippines and Indonesia were unavailable at the time of the download. Sequence data for each country or region underwent cd-hit clustering [69] (threshold, 98.5% identity) to reduce the number of sequences and increase visualization. Countries and regions are defined as in Figure 3. For the calculation, when only the year was available as the isolation date from GISAID, 1 July was used as the tentative isolation date; when only the year and month were available, the 15th was used as the day. This tree was constructed to illustrate the overall evolutionary pathway of Asian IAVs-S, not for precise calculation of the divergence time. Various human seasonal IAVs and IAVs-S from other countries have been included as clade indicators.

In Thailand, viruses with the HA gene of the human seasonal H3N2 virus that circulated during 1996–1997 [21,22] diverged into at least two sub-clades, Thai-a and Thai-b (Figure 4) [22], which have become dominant in Thailand since 2010. Thai-a and Thai-b have replaced the two older human-like lineages—represented, respectively, by A/swine/Udon Thani/NIAH464/2004 and A/swine/Nakhon pathom/NIAH586-1/2005—[19,21,71] which harbored the human seasonal H3 HA that circulated during the early 1970s. Although Thai-a and Thai-b viruses isolated from 2015 through 2017 did not differ antigenically from each other, they differed in antigenicity from the human seasonal H3 viruses isolated during the same period [22]. Descendants of the Thai-b lineage—represented by A/pig/Myanmar/MS-20414/2017—have been reported in Myanmar, suggesting a transborder dissemination of IAVs-S in the region [68].

IAVs-S with the HA gene of cluster IV triple-reassortant (TR) IAVs-S, whose HA gene originated from a human seasonal H3N2 virus that circulated around 1998 in North America [41,75], were isolated thereafter in Korea [55], Vietnam [61], and at an Animal Quarantine Station in Japan [49]. The strain A/swine/Yokohama/aq114/2011 was isolated from the specimens taken from a pig imported from Canada, indicating that the virus entered Japan through pig transportation and that it had not been introduced into the Japanese pig population. In contrast, Korean and Vietnamese IAVs-S in this clade have been isolated on farms in Korea since 2004 and in Vietnam since 2015, thus indicating their establishment in the pig populations of these two countries.

In Taiwan, IAVs-S carrying an HA gene that originated from a human seasonal H3N2 strain isolated around 1980 became established for a decade, from 2002 through 2013. These H3 HA viruses had either N1 or N2 NA genes, indicating reassortments with other IAVs. Given that an ancestral human strain of these Taiwanese IAVs is estimated to have circulated during the late 1970s, the H3HA IAVs-S appeared to have been introduced just once into the Taiwanese pig population. However, whether these or similar strains are still circulating is unknown.

Several strains with an H3 HA from avian influenza viruses or an equine influenza virus have been isolated from pigs in China [47,76,77]. However, these IAVs-S appeared to have resulted from a sporadic incident and have not become established in the pig population of China.

## 4. H5 Subtype IAVs-S

Since 2004, highly pathogenic avian influenza viruses (HPAIVs) carrying an H5 HA gene originated from A/goose/Guangdong/1/96—the so-called Goose/Guangdong (Gs/Gd) lineage—have disseminated throughout the Asian poultry population (Table 2). Concurrently, H5 HPAIVs of the Gs/Gd lineage have sporadically been isolated from pigs in both China [78,79] and Indonesia [80]. In China since 2003, both the H5N1 and H5N6 subtypes were reported [78]. Chinese IAVs-S of the H5N1 subtype belonged to clades 0, 2.3.2.1c, 2.3.4, 5, 7.2, and 9 [78], whereas those of the H5N6 subtype belonged to clade 2.3.4.4 [79]. In Indonesia, H5N1 IAVs-S belonging to clades 2.1.1, 2.1.3 and 2.1.3.3 were isolated from 2005 through 2007. In Korea, an H5N2 IAV-S whose HA gene did not derive from the Gs/Gd lineage but instead belonged to the EA lineage was isolated in 2004 [81].

## 5. H9N2 Subtype IAVs-S

IAVs of H9N2 subtype have been endemic in poultry in Asian countries since the late 1990s [82]. HA genes of this subtype have diversified as the endemic intensified. H9 HA genes have been classified into four major lineages—h9.1, h9.2, h9.3, and h9.4 [83]. The h9.1 and h9.2 lineages are known as the “North American” lineages; the clades h9.3 and h9.4 are further divided into sub-clades indicated with four-digit numerals (e.g., h9.3.3.1, h9.4.1.1). Sub-clades h9.4.1 and h9.4.2 are the so-called the G1- and Y280/G9-lineages, respectively, and h9.3.3 is Y439 or Korean lineage. Sporadic transmission from poultry to swine occurred in China, Hong Kong, and Korea from the late 1990s until 2010. H9N2 IAVs-S from poultry in China and Hong Kong belonged to the h9.4 lineage [83,84,85,86,87,88,89], whereas those in Korea fall into the h9.3, particularly h9.3.3.1 (Table 3). In addition, sequence of Chinese IAV-S belonging to h9.1—A/swine/Yantai/16/2012 (Table 3)—has been reported, suggesting that direct transmission of an AIV directly from a wild bird into Chinese pig population might have occurred.

## 6. IAVs-S of Subtypes Other Than H1, H3, H5, and H9

Owing to intensive surveillance, AIVs have been isolated from pigs in China and Korea (Table 4). Viruses of the subtype H4N1 [91], H4N8 [92], H6N6 [93], H7N9, and H10N5 [94] have been isolated in China and of subtypes H7N2 [95] and H11N6 in Korea. Except for those isolated from specimens collected at slaughterhouses, these viruses were isolated from specimens collected from pigs with respiratory symptoms, including coughing and nasal discharge. These findings indicated that, although viruses of these AIV subtypes caused diseases in infected pigs, they rarely became established in the pig population.

## 7. Discussion

Our current phylogenetic comparison across the IAVs-S isolated from different countries and regions in Asia revealed country- or region-specific evolution in the HA of both the H1 and H3 subtypes of IAVs-S. Among H1 HA subtypes, although clade 1A.3.3.2 viruses derived from human pandemic strains circulated in all of the Asian countries that we compared, co-circulation of other clade(s) of H1 IAVs-S also was evident in most of these countries (Table 1). Co-circulating clades and the relative proportions of each within the H1 subtype vary from one country or region to another. Regarding the H3 HA isolates, although most of them originated from human seasonal H3 IAVs, the clade that predominated differed between Asian countries and regions.

Combining rigorous hygiene practices with a robust IAV-S vaccination program effectively controls IAV-S outbreaks and reduces their economic impact on swine farms. For SI vaccines to be maximally efficacious, the viral strain in the vaccine must be antigenically matched to those circulating on swine farms. In this regard, genetic characterization of IAV-S coupled with their antigenic characterization and followed by timely updates of the vaccine components are essential for each country or region. Differences in antigenicity between IAV-S clades co-circulating in Japanese swine populations have been demonstrated [23]. For example, antiserum produced against the 1A-like virus A/swine/Tochigi/2/2011 (H1N2) reacted eightfold less strongly to A/swine/Yamagata/11/2010 (H1N1) which belongs to clade 1A.3.3.2. Antigenic change among co-circulating clades of a subtype within a country or region or among countries could be accelerated over time, if IAVs-S evolve independently. Should that evolution reaches a point where a vaccine component directed toward one clade no longer offers cross-protection against the other, multiple antigenic components for such subtype need to be included in the vaccine, similar to some vaccines commercially available in North America and Europe [4].

Antigenic change between viruses belonging to the same clade is known to occur within a country or region as well as between them [32]. For example, according to calculation of the mean pairwise antigenic distance (MPD), viruses isolated from Japan and Hong Kong, both belonging to clade 1A as classified by the current criteria, differed by 5.6 antigenic units (AU) [32]. Our current phylogenetic comparison demonstrated that the 1A1.1/1A.1-like isolates from Japan and Hong Kong diverged during the mid-1970s. When such genetic differentiation between countries or regions also increases the antigenic distance, a region- or country-specific SI vaccine is needed.

Human infection by IAVs-S or their reassortants is a threat to public health, as realized by the emergence of H1N1pdm due to reassortment among IAVs-S [96,97,98]. Control of the IAVs-S in pig populations is important not only for preventing the disease burden on pigs but also to prevent the human infection. Incidental human infections with IAVs-S have occurred in North America, Europe, and Australia [99,100,101,102,103,104,105]. Although no large outbreaks in humans have erupted recently in Asian countries, several incidents have been reported in China [46,106,107], Thailand [108], and Vietnam [61]. IAVs-S of the H1 and H3 subtypes are mostly antigenically different from the human seasonal influenza viruses, such that most of the human population lacks protective antibodies against these viruses [109,110,111,112], except for those originating from human H1N1pdm strains. Human infection by IAVs-S has the potential to cause an outbreak and lead to a pandemic, as we have experienced in 2009. Caretakers at farms in Asia are at high risk to IAV-S infection [107,113] and high likelihood of passing that infection to the rest of the country’s population. Effective IAV-S vaccination programs on pig farms could reduce the exposure of humans to IAVs-S and thus the potential risk of a human pandemic [114].

In conclusion, our review of genetic information regarding the HA genes of Asian IAVs-S reveals sparse sequence data from countries where the pig industry is thriving, including Myanmar, the Philippines, India, and Indonesia. Due to a lack of knowledge and the unavailability of effective vaccines, SI is not recognized as a major swine disease by pig producers in these countries. Therefore, most Asian countries neither monitor circulating IAVs-S nor vaccinate against IAV-S routinely. Furthermore, our findings reveal geographical segregation of the genetic evolution of the HA genes within the subtypes. This genetic segregation points to the need to develop region- or country-specific IAV-S vaccines for their maximum efficacy. However, genetic segregation is not always directly correlated with antigenic segregation. Therefore, the antigenic diversity of IAVs-S needs to be evaluated concurrently with available genetic diversity data to develop vaccines that are well matched to—and thus highly protective against—the IAVs-S circulating in the swine populations in each country or region in Asia. Whether such an approach is feasible remains to be seen.

## Figures and Tables

**Table 1:** Prevalence of each clade of IAV-S H1 isolates in each Asian country since 2010.

Country or Region	Total No. of Sequences in GISAID ^a^	Total No. of Sequences after Cd-Hit (99.5% Identity) ^b^	Proportion (%) of Each Clade among H1 IAV-S H1 Since 2010 (%) ^c^												
			1A.1/1A.1-like	1A.1.1	1A.1.2	1A.1.3	1A.2	1A.3.3	1A.3.3.1	1A.3.3.2	1A.3.3.3	1B.2	1C.1-2-like	1C.2	1C.2.3
China	317	134	1.4			3.0			1.5	11.9					82.1
Hong Kong	3	3								33.3					66.7
India	1	1								100.0					
Japan	473	119	56.3							42.9	0.8				
Korea	16	8					12.5			50.0	25.0				12.5
Myanmar	4	2			50.0					50.0					
Taiwan	9	8		37.5						62.5					
Thailand	138	29	3.4		24.1					69.0			3.4		
Vietnam	199	28						32.1		42.9		21.4		3.6	

^a^ Sequences of isolates that were isolated after 2010 and contained the entire HA coding region were counted. ^b^ Sequences more than 99.5% identical were clustered by using cd-hit [69]. To avoid overestimation of a particular clade due to the prevalence derived from a single farm or outbreak, a single representative sequence for each cluster underwent prevalence analysis because inclusion of all the sequences available in the database may result in overestimation. The 99.5% identity threshold was set empirically. Sequences remaining after using cd-hit were classified according to clade; the number of sequences in each clade was divided by the total number of sequences remaining after cd-hit clustering to calculate each clade’s percentage. ^c^ Clade classification was done by using the Swine H1Clade Classification Tool at Influenza Research Database (IRD: https://www.fludb.org/brc/h1CladeClassifier.spg?method=ShowCleanInputPage&decorator=influenza, accessed on 30 July 2021).

**Table 2:** H5 IAVs-S isolated in Asian countries.

Representative Strain from Each Cluster ^a^	Isolation Date ^b^	Subtype	Country	Clade ^c^	GISAID Isolate ID	Reference
A/swine/Shandong/2/03	2003	H5N1	China	0	EPI_ISL_4138	none available
A/swine/Banten/UT2071/2005	2005	H5N1	Indonesia	2.1.1	EPI_ISL_76964	[80]
A/swine/Tabanan/061/2006	30 September 2006	H5N1	Indonesia	2.1.3	EPI_ISL_190517	none available
A/swine/Banten/UT6008/2007	2007	H5N1	Indonesia	2.1.3	EPI_ISL_76972	[80]
A/swine/East_Java/UT6010/2007	2007	H5N1	Indonesia	2.1.3.3	EPI_ISL_76973	[80]
A/swine/Korea/C12/2008	2008	H5N2	Korea	EA-nonGsGD	EPI_ISL_28756	[81]
A/swine/Jiangsu/1/2008	December 2008	H5N1	China	7.2	EPI_ISL_144531	[78]
A/swine/Jiangsu/2/2009	January 2009	H5N1	China	2.3.4	EPI_ISL_144532	[78]
A/swine/Guangdong/2/2014	18 June 2014	H5N6	China	2.3.4.4	EPI_ISL_196101	[79]
A/swine/Shandong/SD1/2014	14 October 2014	H5N1	China	5	EPI_ISL_226073	none available
A/swine/Shandong/SD2/2014	3 November 2014	H5N1	China	9	EPI_ISL_226074	none available
A/swine/Zhejiang/SW57/2015	January 2015	H5N1	China	2.3.2.1c	EPI_ISL_393329	none available
A/swine/Guangdong/G3/2015	26 March 2015	H5N6	China	2.3.4.4	EPI_ISL_266596	none available

^a^ Sequences found in GISAID, which contains the entire open reading frame of the HA protein of isolates from each country, separately underwent cd-hit clustering (threshold, 98.5% homology); the most recent isolate in each cluster is listed as the representative strain. ^b^ Isolation date is given as Day Month Year, unless the month or date (or both) was unavailable. ^c^ Clade classification was performed at https://www.fludb.org/brc/h5n1Classifier.spg?method=ShowCleanInputPage&decorator=influenza on 11 August 2021.

**Table 3:** H9 IAV-S isolated in Asian countries.

Representative Strain from Each Cluster ^a^	Isolation Date ^b^	Subtype	Country	Clade ^c^	GISAID Isolate ID	Reference
A/swine/Hong Kong/10/1998	1998	H9N2	Hong Kong	h9.4.2.3	EPI_ISL_379214	[90]
A/swine/ShanDong/1/2003	2003	H9N2	China	h9.4.2.3	EPI_ISL_3465	[87]
A/swine/Guangxi/58/2005	2005	H9N2	China	h9.4.2.3	EPI_ISL_12580	[84]
A/swine/Jiangxi/1/2004	2004	H9N2	China	h9.4.2.4	EPI_ISL_15583	[84]
A/swine/Korea/S452/2004	2004	H9N2	Korea	h9.3.3.1	EPI_ISL_4617	none available
A/swine/Guangxi/7/2007	1 February 2007	H9N2	China	h9.4.2.4	EPI_ISL_81608	[84]
A/swine/Henan/Y1/2009	2 July 2009	H9N2	China	h9.4.2.5	EPI_ISL_139172	[88]
A/swine/Shanghai/Y1/2009	13 October 2009	H9N2	China	h9.4.2.5	EPI_ISL_139173	none available
A/swine/Guangdong/L1/2010	30 January 2010	H9N2	China	h9.4.2.1	EPI_ISL_179451	[89]
A/swine/Yantai/16/2012	21 September 2012	H9N2	China	h9.1	EPI_ISL_229212	none available
A/swine/China/SPF_embryonated_chicken_eggs/2015	18 May 2015	H9N2	China	h9.4.2.5	EPI_ISL_381335	none available
A/swine/Shandong/TA009/2019	April 2019	H9N2	China	h9.4.2.5	EPI_ISL_503942	none available

^a^ Sequences found in GISAID, which contains the entire open reading frame of the HA protein of isolates from each country, separately underwent cd-hit clustering [69] (threshold, 98.5% homology); the most recent isolate in each cluster was listed as the representative strain. ^b^ Isolation date is as Day Month Year, unless the month or date (or both) was unavailable. ^c^ Clade classification follows Jiang, et al., 2012.

**Table 4:** IAVs-S of subtypes other than H1, H3, H5, and H9 subtypes isolated in Asian countries.

Strain ^a^	Isolation Date ^b^	Subtype	Country	GISAID Isolate ID	Reference
A/swine/HuBei/06/2009	24 May 2009	H4N1	China	EPI_ISL_130351	[91]
A/swine/Guangdong/K4/2011	21 October 2011	H4N8	China	EPI_ISL_127501	[92]
A/swine/Yangzhou/080/2009	January 2009	H6N6	China	EPI_ISL_139117	none available
A/swine/Guangdong/K6/2010	17 January 2010	H6N6	China	EPI_ISL_89164	[93]
A/swine/KU/16/2001	2001	H7N2	Korea	EPI_ISL_85566	[95]
A/swine/eastern_China/005/2017	20 August 2017	H7N9	China	EPI_ISL_505059	none available
A/swine/eastern_China/HH24/2017	7 August 2017	H7N9	China	EPI_ISL_505060	none available
A/swine/Hubei/10/2008	30 April 2008	H10N5	China	EPI_ISL_129071	[94]
A/swine/KU/2/2001	2001	H11N6	Korea	EPI_ISL_80217	none available

^a^ IAVs-S from Asian countries found in GISAID, which contains the entire open reading frame of each HA protein. ^b^ Isolation date is given as Day Month Year, unless the month or date (or both) was unavailable.
